# Predicting the side effects of influenza vaccination

**DOI:** 10.1093/abm/kaaf024

**Published:** 2025-03-26

**Authors:** Connor Silvester, Chiara Gasteiger, Greg D Gamble, Marc S Wilson, Kate Faasse, Keith J Petrie, Kate MacKrill

**Affiliations:** Department of Psychological Medicine, University of Auckland, New Zealand; Psychology and Neuroscience, Auckland University of Technology, Auckland, New Zealand; Department of Psychological Medicine, University of Auckland, New Zealand; Department of Psychology, Stanford University, United States; Department of Medicine, University of Auckland, New Zealand; School of Psychology, Victoria University of Wellington, New Zealand; School of Psychology, University of New South Wales, Australia; Department of Psychological Medicine, University of Auckland, New Zealand; Department of Psychological Medicine, University of Auckland, New Zealand

**Keywords:** anti-vaccination, influenza, nocebo, side effects, vaccination

## Abstract

**Background:**

Side effects following vaccination intensify vaccine hesitancy, which remains a significant challenge to public health. Research suggests that a proportion of side effects are not caused by the vaccine but are instead associated with psychological factors that influence nocebo responding.

**Purpose:**

This study investigates the psychological and demographic factors associated with symptom reporting postvaccination, the attribution of these symptoms as side effects, and their influence on future intentions to vaccinate.

**Methods:**

A prospective, longitudinal design was employed with 225 influenza vaccination recipients. Demographic and psychological measures (including anxiety, vaccination attitudes, and side effect expectations) were completed at baseline. Side effects were measured immediately and 1-week following the vaccination. Future intentions to vaccinate were measured 1-week postvaccination.

**Results:**

Anxiety (*P* < .001) and perceived sensitivity to vaccines (*P* = .044) predicted the number of symptoms reported immediately following vaccination. Anxiety (*P* < .001) and perceived sensitivity (*P* = .035) along with baseline symptoms (*P* < .001) predicted symptoms 1 week following the vaccination. Female gender (*P* = .003), younger age (*P* = .018), anxiety (*P* < .001), and baseline symptoms (*P* = .009) predicted whether participants attributed symptoms as vaccination side effects. Anti-vaccination attitudes were associated with less intention to vaccinate in the future (*P* = .033).

**Conclusions:**

Nocebo-associated psychological factors contributed to symptoms experienced after an influenza vaccination. Findings demonstrate that the way symptoms are noticed, and then interpreted as side effects, appear to be separate mechanisms promoted by different factors. This study improves identification of side effect reporters prior to vaccination.

## Teaser

Psychological factors predict the number of symptoms people notice after a flu vaccination, while demographic factors influence whether they believe these are side effects of the vaccine.

## Introduction

Vaccine hesitancy is a growing threat to public health. Although there is substantial evidence that vaccination has controlled various communicable diseases, negative attitudes, and misconceptions relating to safety and efficacy can undermine the public health benefit of vaccination.^1^ Vaccine hesitancy is a significant problem in managing novel global outbreaks, such as COVID-19 and preventing re-emerging outbreaks of previously controlled diseases.^2^

The experience of physical symptoms following vaccination is a common factor driving vaccine hesitancy. Despite being low-risk, routine vaccines such as the annual influenza vaccination, are often associated with transient, benign side effects.^3^ Research suggests that medication side effects are not always associated with the active ingredients of a medication. The nocebo effect describes the tendency for individuals to experience side effects that cannot be attributed to the medication but rather negative expectations for harm.^3^ This phenomenon has been shown to cause some of the adverse responses to vaccination.^4^ Therefore, it is often the anticipation of vaccine side effects that can decrease vaccination intention, contribute to the development of anti-vaccination attitudes, and ultimately increase healthcare utilization.^5–7^

A recent review suggests that past research has often oversimplified the mechanistic pathway of the nocebo effect.^8^ The typical perception is that negative expectations directly lead to nocebo side effects but this has been criticized for neglecting the intermediatory influence of symptom attention and symptom attribution.^9^ A study by Geers and colleagues showed that expectations alter the type of somatic information that an individual attends to and how they (mis)interpret it.^10^ In this experiment, 1 group was deceptively told a placebo tablet contained caffeine, with a double-blind group told it may or may not contain caffeine. Participants in both these groups reported attending more closely to caffeine-related physical sensations but the deceptive group went on to report more caffeine-related sensations. This suggests the processes of symptom awareness and symptom attribution are not necessarily linear and could therefore be affected by different factors.

We propose that the distinction between symptom attention and symptom misattribution is essential for accurately identifying the nocebo effect. In regards to vaccination, it is likely that the act of vaccination cues an individual to focus their attention on their bodily signals, prompting increased awareness of their transient and benign physical sensations.^4,11^ These common, everyday symptoms are then misinterpreted as side effects of the vaccine.^12–14^ Actively separating these 2 processes in the nocebo effect might better delineate the factors that promote side effect reporting and inform interventions to reduce nocebo responding. This study investigates symptom reporting following influenza vaccination and aims to identify the psychological and demographic factors that contribute to the awareness of symptoms and the attribution of these as side effects.

## Methods

### Study design and participants

This study was a prospective cohort study, with 3 assessment points (baseline, immediately after vaccination, and 1-week follow-up). Ethics approval was obtained from the University of Auckland Human Participants Ethics Committee (reference 022819). The study method and results are reported following Strengthening the Reporting of Observational Studies in Epidemiology (STROBE) Statement.

A sample size of 205 was required using a power level of .80, an alpha level of .05, and a small to medium effect (*f*^2^ = .08) based on previous studies exploring psychological variables on side effect reporting and attribution.^5,6,14–16^ To allow for attrition of approximately 10%, it was determined that 225 participants should be recruited.

### Procedure

Students who received the influenza vaccination in April and May of 2019 at Victoria University of Wellington in New Zealand were recruited. Participants were eligible if they were over the age of 18 and fluent in English. Participants were excluded from the study if they were pregnant or breastfeeding, had a history of adverse reactions to influenza vaccinations, had already received the 2019 influenza vaccination, were unable to give consent, or were determined ineligible by staff administrating the vaccine.

The study was advertised on university-associated social media as an investigation into the side effects of influenza vaccinations. Potential participants were informed that the study aimed to understand individual differences in side effects following an influenza vaccination. At the vaccination sites, interested students approached the investigators and were given a participant information sheet and screened for eligibility. No attempt was made to correct for potential bias from convenience sampling. After eligibility was confirmed and informed consent obtained, participants were provided a baseline questionnaire assessing demographics, side effect expectations, anxiety, vaccination attitudes, perceived sensitivity to vaccines, and baseline symptom experience—variables identified in past research as predictors of the nocebo effect and side effect reporting.^17^ Upon completion, vaccination staff administered the trivalent inactivated influenza vaccination and monitored participants for adverse reactions for 20 minutes. Following this period, participants completed the immediate follow-up questionnaire, which assessed side effects experienced since receiving the vaccination. Seven days following vaccination, a final follow-up questionnaire was emailed to participants to investigate symptoms experienced since the vaccination, whether participants believed these were vaccine side effects, and their future vaccination intention. Participants were offered a $20 shopping voucher for taking part in the study. They received a written debriefing form on completion of the study explaining the full aim; that the study was interested in associations between side effects and individual factors, such as anxiety and anti-vaccination beliefs. Participants were given the opportunity to withdraw their data after receiving this information, which none did.

**Figure 1.**
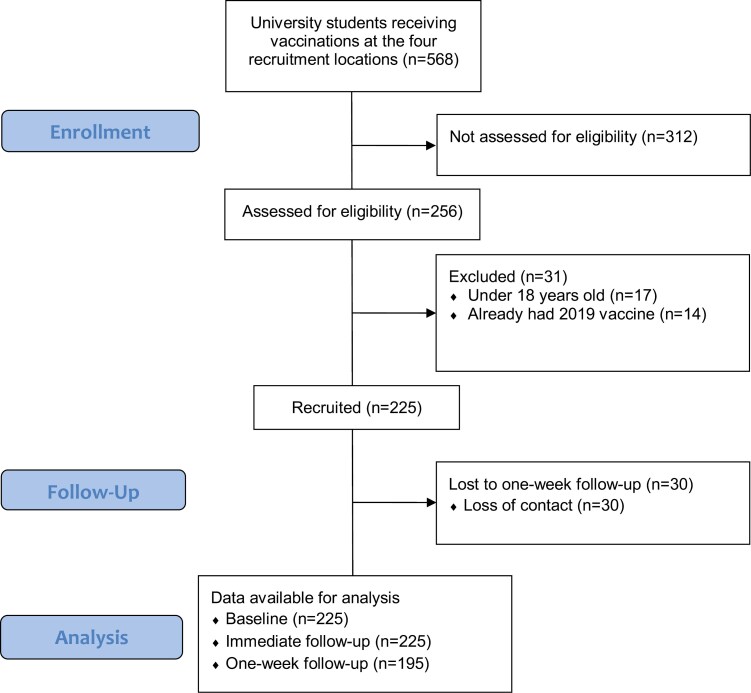
STROBE diagram showing participant progression within the study. University students receiving vaccinations at the 4 recruitment locations (*n* = 568).

Data was collected between 9 April and 7 May 2019. A total of 568 students received influenza vaccinations at vaccination sites where data were collected. Of these individuals, 256 approached the researcher for participation in the study. Seventeen potential participants were excluded as they were younger than 18, while a further 14 had already received the 2019 influenza vaccination. In total, 225 individuals were eligible and consented to participation (Figure 1). All 225 individuals completed the baseline questionnaire and the immediate follow-up questionnaire. Of these participants, 195 (87%) completed the 1-week follow-up assessment.

### Baseline measures (predictor variables)

#### Demographics

Participants provided information about their age, gender, and ethnicity. Information regarding participants’ socioeconomic status was not collected.

#### Baseline symptom experience

Participant symptom experience was measured using the Side Effect Attribution Scale (SEAS).^18^ This 2-part symptom measure comprises 50 items identified as common side effects across various medications. For baseline symptoms, part 1 of the SEAS was administered, which asked participants whether they had experienced each of the 50 symptoms in the previous 4 weeks with the response options of “Yes” or “No.” A total symptom score is created by summing “Yes” responses. One item assessing anxiety was removed, due to a validated anxiety measure being used as a predictor variable.

#### Perceived sensitivity to vaccination

The Perceived Sensitivity to Medicines Scale (PSM) assessed how sensitive individuals believed they were to vaccination effects by replacing the term “medicine” with “vaccination.”^19^ One item of the PSM did not translate into a vaccination context (“Even small amounts of medicines can upset my body”) and so was removed. Participants responded on a 5-point Likert scale, with higher total scores indicating a greater perceived sensitivity to vaccination.

#### Anxiety

Participants completed the 6-item, short-form Spielberger State-Trait Anxiety Inventory (STAI-SF) to assess trait anxiety.^20^ Participants responded on a 4-point Likert-type format with higher total scores indicating higher trait anxiety.

#### Vaccination attitudes

To assess anti-vaccination attitudes, participants completed the 12-item Vaccination Attitudes Examination Scale (VAX).^1^ Items include “I feel safe after being vaccinated” to which participants rate their level of agreement using a 6-point Likert-type format. Higher total scores reflected stronger anti-vaccination attitudes.

#### Side effect expectations

One item adapted from vaccination literature assessed expectations about experiencing side effects following the vaccination.^5,21^ Participants answered using a 10-point Likert scale with higher scores indicating greater side effect expectations.

### Immediate follow-up measures (outcome variable)

#### Symptoms immediately following vaccination

A 14-item scale was constructed from the local and systemic adverse events occurring in individuals who received the 2017 influenza vaccination, as listed in the New Zealand Data Sheet for the 2017 FluVax. Participants indicated if they had experienced each of the 14 symptoms following their vaccination and, where applicable, the severity as either mild, moderate or severe. Responses were collated by creating a total symptom score and an average severity rating across the symptoms. This measure was used as it assessed specific vaccine-related symptoms that participants were more likely to experience during the post-vaccination wait time, compared to the other more general symptoms assessed by the SEAS that would be difficult for participants to notice in this timeframe (eg, sleep problems and changes in appetite).

### One-week follow-up measures (outcome variables)

#### Symptoms 1-week following vaccination

Part 1 of the SEAS was administered again to assess symptoms experienced in the 7 days following the vaccination.^18^ Part 2 was administered to assess whether participants interpreted their symptoms as side effects from the vaccination. For each symptom experienced, participants were asked to rate on a 5-point scale the degree to which they thought it was caused by the vaccination, ranging from 1 “Definitely not a side effect” to 5 “Definitely a side effect.” Responses of 3 “Unsure” or greater were coded as an attribution of the symptom to the vaccination and therefore a vaccination side effect.

The symptoms of the SEAS were further categorized as either influenza-specific or influenza nonspecific. Specific symptoms were those that aligned with the clinical diagnostic criteria for influenza, while nonspecific influenza symptoms were those that did not align with the criteria. Eight symptoms were characterized as specific symptoms (headache, muscle pain, runny or blocked nose, cough, fatigue, drowsiness, sore throat, and fever), while the remaining 41 were considered nonspecific.

#### Future intention to vaccinate

One item assessed the participant’s intention to be vaccinated in the next vaccination season. Participants responded on a 5-point Likert scale ranging from 1 “Strongly disagree” to 5 “Strongly agree.” Scores higher than 3 “Neither agree nor disagree” were recorded as an intention to be vaccinated again (1), while scores 3 and below were coded as no intention to be vaccinated (0).

### Statistical analysis

The data was analyzed using IBM SPSS v29, with statistical significance taken at *P* < .05. If an item value was missing for a scale total, this point was replaced with the average of the other items rounded to the nearest whole number. Participants who lost to the 1-week follow-up were removed from these analyses.

Descriptive analyses were conducted for sample characteristics. Pearson correlations were conducted to investigate the initial associations between the baseline and outcome variables. Generalized linear regression models were conducted to investigate whether the baseline variables predicted the outcome variables immediately and 1 week after vaccination. The predictor variables were age, gender, side effect expectations, anxiety, vaccination attitudes, perceived sensitivity to vaccines, and baseline symptom experience. Outcome variables were the total number of symptoms reported immediately following vaccination, the severity of immediate symptoms, the total number of symptoms reported 1 week post-vaccination, 1-week symptoms attributed to the vaccination, and number of specific and nonspecific symptoms attributed to the vaccine. Outcome variables that were count data, which are usually not normally distributed, were analyzed using a Poisson distribution and log link function, while all other analyses used a normal distribution and identity link function. A logistic regression was conducted to investigate whether the baseline variables predicted participants’ intention to vaccinate in the future.

## Results

The sample was predominantly female (73%) with a mean age of 19 years (*SD* = 2.6). Most were New Zealand European (74%) or Māori (10%). Only a small group (23%) received the flu vaccine in the past year, likely due to the cost associated with vaccination in the community whereas it was free through the university. At baseline, there was a total of 2428 symptom reports, with participants experiencing an average of 11 (*SD* = 6.4) symptoms in the last month. On average participants had low baseline scores for side effect expectations, anti-vaccination attitudes, anxiety, and perceived sensitivity to vaccines (Table 1). Attrition analyses revealed that those who did not complete the 1-week follow-up had significantly higher anti-vaccination scores (*M* = 30.6, *SD *= 6.0) than those who did complete the follow-up (*M* = 27.2, *SD* = 9.0), *t*(223) = 2.10, *P* = .034. See Table 2 for the initial correlations between baseline and outcome variables and Table 3 for the full regression analysis results.

**Table 1:** Demographic information of participants (*N* = 225).

Variable	*M* ± *SD* (range) *N* [%]
Age (years)	18.7 ± 2.6 (18-46)
Gender	
Male	60 [26%]
Female	165 [73%]
Ethnicity	
New Zealand European	167 [74%]
Māori	23 [10%]
Pacific islands	7 [3%]
Asian	14 [6%]
Other European	7 [3%]
Other	7 [3%]
Flu vaccine last year	50 [23%]
Number of flu vaccines (past 5 years)	1.7 ± 1.5 (0-5)
Doctor visits (past 12 months)	3.7 ± 4.1 (0-50)
Number of symptoms (past month)	10.3 ± 6.2 (1-33)
Side effect expectations	3.5 ± 2.3 (1-10)
Anxiety	11.9 ± 3.6 (6-24)
Vaccination attitudes	27.7 ± 8.2 (12-53)
Perceived sensitivity to vaccines	6.5 ± 3.0 (4-17)

**Table 2:** Correlation table for associations between predictor and outcome variables.

	Immediate side effect total	Immediate side effect severity	1-Week symptom total	1-Week symptoms attributed to vaccine	Specific flu symptoms attributed to vaccine	Nonspecific symptoms attributed to vaccine	Intention to vaccinate in future
Age	.03	–.05	–.07	–.09	–.09	–.04	.07
Gender	–.00	.10	.05	.15*	.11	.10	.01
Side effect expectations	.10	.24**	.07	.14	.18**	.15*	–.18*
Anxiety	.22***	.04	.28***	.11	.14*	.12	–.03
Vaccination attitudes	–.05	.14	.01	.08	.10	.17	.20**
Perceived sensitivity to vaccines	.14*	.18*	.07	.07	.14*	.23	–.04
Baseline symptoms	.13	.07	.25***	.12	.09	.08	.01

*P < .05, **P < .01, ***P < .001. Gender coded as 0 = male, 1 = female.

**Table 3:** Generalized linear model regression results for baseline predictors and outcome measures.

	Immediate side effect total		Immediate side effect severity		1-Week symptom total		1-Week symptoms attributed to vaccine		Specific flu symptoms attributed to vaccine		Nonspecific symptoms attributed to vaccine		Intention to vaccinate in future	
Predictor	B (SE)	*P*	B (SE)	*P*	B (SE)	*P*	B (SE)	*P*	B (SE)	*P*	B (SE)	*P*	B (SE)	*P*
Age	.02 (.02)	.38	–.01 (.02)	.65	–.02 (.02)	.21	–**.18 (.08)**	**.018**	–.12 (.07)	.08	–.02 (.04)	.69	.43 (.43)	.32
Gender	–.15 (.11)	.17	.05 (.08)	.52	–.02 (.08)	.75	**.59 (.20)**	**.003**	.22 (.20)	.26	.29 (.21)	.16	.38 (.60)	.52
Side effect expectations	.04 (.02)	.11	**.04 (.02)**	**.032**	.01 (.02)	.55	.07 (.03)	.06	**.09 (.04)**	**.016**	**.09 (.04)**	**.019**	–.13 (.12)	.28
Anxiety	**.06 (.01)**	**<.001**	.00 (.01)	.96	**.06 (.01)**	**<.001**	**.07 (.02)**	**<.001**	**.07 (.02)**	**.001**	**.09 (.02)**	**<.001**	–.06 (.08)	.50
Vaccination attitudes	–.01 (.01)	.11	.01 (.00)	.68	.00 (.00)	.96	.02 (.01)	.11	.01 (.01)	.52	.02 (.01)	.13	–**.08 (.04)**	**.033**
Perceived sensitivity to vaccines	**.03 (.02)**	**.044**	.02 (.01)	.15	**.02 (.01)**	**.035**	.01 (.02)	.75	.04 (.03)	.11	.03 (.03)	.25	–.01 (.09)	.91
Baseline symptoms	.01 (.01)	.48	.00 (.01)	.84	**.02 (.01)**	**<.001**	**.03 (.01)**	**.009**	.00 (.01)	.73	.02 (.01)	.21	.02 (.05)	.68

Gender coded as 0 = male, 1 = female.

### Immediate symptoms

#### Symptom reporting

Immediately following the vaccination, 170 participants (76%) reported experiencing at least 1 symptom, with the average number being 2.0 symptoms per person (*SD* = 2.0). Of the 14 symptoms assessed, injection site pain, muscle aches, redness at the injection site, light-headedness, and swelling at the injection site were the most frequently reported. The generalized linear model with a Poisson distribution investigating predictors of symptom reporting was significant (likelihood ratio χ^2^ (7) = 37.68, *P* < .001, McFadden pseudo-*R*^2^ = .171). Higher levels of anxiety and perceived sensitivity to vaccines significantly predicted greater symptom totals postvaccination.

#### Symptom severity

The average symptom severity rating was 1.3 (*SD* = .4) indicating only mild severity. The overall model for predictors of symptom severity was not significant (Likelihood ratio χ^2^ (7) = 13.13, *P* = .069, McFadden pseudo-*R*^2^ = .011) but despite this, greater side effect expectations were a significant predictor of severity ratings.

### One-week follow-up

#### Symptom reporting

**Figure 2.**
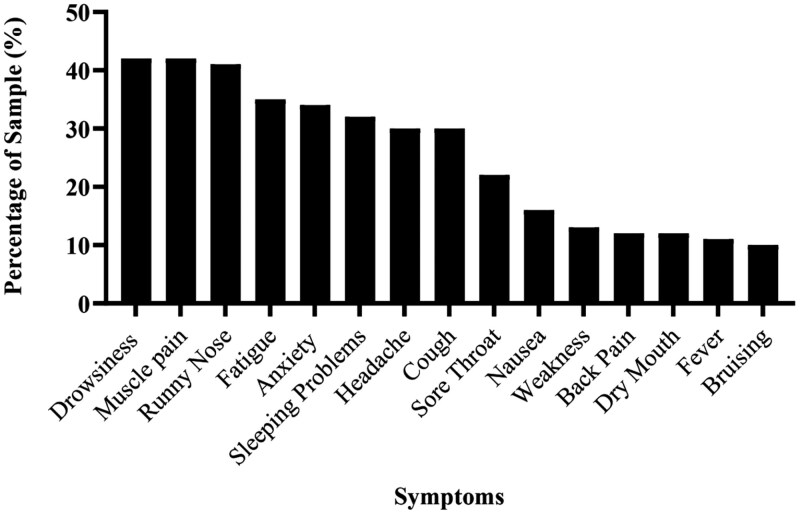
The 15 most frequent symptoms reported at 1-week follow-up.

One week following the influenza vaccination, participants reported an average of 4.6 (*SD *= 4.5) symptoms, with drowsiness, muscle pain, and runny nose being the most common (see Figure 2). One hundred and fifty-five participants (80%) reported experiencing at least 1 symptom. Of the 2428 symptoms reported at baseline, 4.7% (114) went on to be attributed to the vaccine at the 1 week follow-up. The most frequently attributed baseline symptoms were characteristic of the influenza virus or vaccination: muscle pain (attributed by 9.3% of the sample at 1 week follow-up), headache (4.9%), fatigue (4.9%), runny or blocked nose (4.4%), and cough (4.4%).

The generalized linear model with a Poisson distribution explored whether baseline variables predicted symptom reporting was significant (likelihood ratio χ^2^ (7) = 97.94, *P* < .001, McFadden pseudo-*R*^2^ = .073). Higher levels of anxiety, perceived sensitivity to vaccines, and baseline symptom experience were significantly associated with reporting more symptoms.

#### Symptom attribution

Of the symptoms experienced in the week following vaccination, an average of 1.2 (*SD* = 2.5) were attributed to the vaccine as side effects. Only 41 participants (26%) who experienced at least 1 symptom did not attribute them to the vaccine. The generalized linear model with Poisson distribution analysis was significant (likelihood ratio χ^2^ (7) = 66.97, *P* < .001, McFadden pseudo-*R*^2^ = .224). Significant predictors of side effect attribution were younger age, female gender, higher anxiety, and experiencing a greater number of baseline symptoms.

#### Specific and nonspecific symptom reporting

Participants attributed an average of 0.84 (*SD* = 1.5) specific symptoms and 0.78 (*SD* = 1.8) nonspecific symptoms as side effects from the vaccine. The generalized linear model was significant for both specific symptoms (Likelihood ratio χ^2^ (7) = 40.58, *P* < .001, McFadden pseudo-*R*^2^ = .161) and nonspecific symptoms (Likelihood ratio χ^2^ (7) = 46.11, *P* < .001, McFadden pseudo-*R*^2^ = .173). In both models, greater side effect expectations and higher anxiety significantly predicted a greater tendency to attribute specific and nonspecific symptoms to the vaccine.

#### Future intention to vaccinate

After receiving the influenza vaccination, 176 participants (91%) intended to receive the vaccination again in the following year. The logistic regression analysis investigating baseline predictors of intention was not significant (χ^2^ (7) = 12.91, *P* = .074, McFadden pseudo-*R*^2^ = .114). However, greater endorsement of anti-vaccination attitudes was a significant predictor of lower intention to vaccinate in the future, with an odds ratio of 0.9, 95% confidence interval [0.86, 0.99].

## Discussion

The results from this study suggest that known predictors of the nocebo effect do contribute to a proportion of symptoms experienced following influenza vaccination. Higher anxiety and perceived sensitivity to vaccines significantly predicted the number of symptoms reported immediately after vaccination, with anxiety being the only predictor of side effect severity. Additionally, anxiety, perceived sensitivity to vaccines and symptoms experienced at baseline predicted symptom reporting 1 week post vaccination. However, it was anxiety, baseline symptoms plus the demographic factors of younger age and female gender that were associated with whether these symptoms were then attributed as vaccine side effects. When breaking down the attribution of specific flu symptoms or nonspecific symptoms to the vaccine, both were predicted by anxiety and side effect expectations. Finally, a higher anti-vaccination attitude was the only variable associated with a decreased intention to vaccinate in the future. However, the pseudo-*R*^2^ values indicate that there is still a large amount of outcome variance unexplained by the model predictors.

This study is one of the first to identify the different characteristics that influence 2 distinct mechanisms that promote the nocebo effect: (1) how symptoms are noticed; and (2) how they are then attributed to a vaccine as side effects. Similar across both processes is the effect of anxiety and baseline symptom experience. However, the cognitive variable of perceived sensitivity to vaccines influenced symptom awareness, while the demographic factors of age and gender affected symptom attribution.

These findings are supported by past research. Anxiety and perceived sensitivity to vaccines are factors known to be associated with side effect reporting due to their influence on bodily attention and misattribution processes. As per the symptom-perception hypothesis, negative affect firstly influences attention toward bodily sensations.^3,22^ Secondly, anxiety and perceived sensitivity promote more negative interpretations, prompting the misattribution of ambiguous physical sensations to an external cause, such as illness or medical intervention.^3,4,14–16,23,24^ However, a recent meta-analysis has broken down the different types of anxiety, finding that state anxiety is a stronger predictor of nocebo effects compared to trait anxiety, which is not associated with the magnitude of the nocebo effect.^25^ This suggests that anxiety-inducing factors inherent to a situation, such as receiving information about side effects or the act of being vaccinated, are more influential on side effect reporting than an individual’s dispositional anxiety levels.

Interestingly past research has found inconclusive evidence for the role of gender and age in nocebo effects.^17^ In a study on predictors of side effects from a generic antidepressant, older age was associated with increased reporting,^26^ whereas in the placebo arm of an olanzapine trial, younger participants reported more side effects.^27^ It is typically shown that females report more symptoms and are more susceptible to anxiety than males, however, the effect of this on nocebo responses may be influenced by the way expectations are induced. It has been suggested that females exhibit a greater nocebo response through learning/conditioning paradigms, while males are more susceptible to verbally induced symptom provocation.^28^ The setting of the current study did not provide an explicit side effect expectation manipulation and therefore may have encouraged greater recall of past vaccination experiences, thereby producing the effect seen with the female gender.

Both side effect expectations and anxiety predicted specific and nonspecific symptom attribution, suggesting that there is not a separate process depending on the type of symptom. This may explain why both symptoms associated with flu (eg, cough, runny nose, and headache) and other transient symptoms (eg, nausea and weakness) feature in the 15 most common symptoms reported following vaccination. Surprisingly, greater expectations for side effects uniquely predicted this type of reporting but were not associated with total side effects. It may be that the questionnaire itself, which provided a list of symptoms, primed participants to attend to their bodily sensations and notice a range of symptoms beyond the typical markers of vaccination/flu, and therefore inflated their symptom reporting.^29^

Anti-vaccination attitudes have been strongly associated with side effect reporting in previous literature^14,16,30^ but made no significant contributions in the attribution of symptoms as vaccine side effects in the current study. These inconsistencies may be associated with the nature of the sample. The sample consisted of individuals willing to receive the vaccination and as such the variance of anti-vaccination attitudes is likely lower than the general population, limiting the explanatory power in regressions conducted. Nevertheless, anti-vaccination attitude was the sole predictor of intentions to vaccinate in the future, which is a common relationship seen in other studies.^1^

The findings from this study have clinical and theoretical implications. Clinically, the results indicate how psychological variables contribute to symptom experience following vaccination. Findings may represent a diagnostic tool for identifying people more likely to report side effects following vaccination based on their baseline psychological characteristics. While this study was conducted prior to the COVID-19 pandemic, the level of vaccine hesitancy and anti-vaccination attitudes in the general population has likely significantly increased. The modest relationships identified in this study are probably stronger and more influential in the post-COVID environment, for example, concerns about side effects and the short development period were identified as major barriers to COVID-19 vaccine acceptance.^31,32^ Understanding individuals’ expectations, beliefs, and anxiety will allow the tailoring of health information to ensure the uptake of novel vaccines in the future. Interventions could include passive measures (eg, calming environments, utilizing distraction techniques to reduce attention to bodily sensations, and increasing patient privacy) or active measures (eg, anxiety management and altering vaccination information).

The study also has theoretical implications for how nocebo responses are measured. Conceptually, the attribution mechanism is often overlooked, and instead, a focus is provided on measuring symptoms reported in response to a medication.^29,33^ Symptom attribution needs to be assessed in tandem with symptom experience to fully capture the nocebo effect. Previous scales often produce data with low reliability and fail to discriminate between different individuals’ beliefs.^34^ Future research should employ measurements, such as the Side Effect Attribution Scale, that systematically discriminate nocebo mechanisms.^18^

The study has the strength of a relatively good sample size and retention rate at follow-up as well as all participants receiving a vaccine under standardized conditions. However, the limitations of the study should be considered. Firstly, the sample was predominantly drawn from a younger, university-educated population already engaging in influenza vaccinations. Therefore, findings may be limited in generalizability and are unlikely to extend to those with strong anti-vaccination views who would not present for vaccines. Similarly, almost 3-quarters of the sample was female, which may have artificially inflated the strength of the variable relationships seen as symptom reporting and anxiety disproportionately impact women.^35^ Secondly, as vaccines are pharmacologically active substance, it is difficult to disentangle symptoms caused by the treatment and those that are nocebo responses. However, the fact that side effects were associated with known predictors of the nocebo effect suggests that a nocebo component was present. Research employing placebo-controlled designs or utilizing biomarkers could confirm physiological versus nocebo causes of symptoms. Additionally, the study utilized different measures of symptom reporting immediately postvaccination and 1-week later. This may impact the ability to compare predictors of side effect attribution at these time points.

There are several avenues for future research as a result of this study. Future research should investigate whether implementing nocebo-minimizing therapies, such as a nocebo explanation, positive framing, and targeting side effect mindsets, can reduce reporting of vaccine side effects.^36–38^ Should a randomized controlled trial reveal that these interventions are effective, they may present a feasible, cost-effective intervention that can be delivered briefly before vaccinations are received. However, these interventions need to be tested in samples with higher anti-vaccination attitudes and methods developed to retain such individuals in these studies. Future research may also benefit from measuring predictor variables before and after immunization, to investigate whether specific vaccination experiences shape psychological well-being and vaccine perceptions. Similarly, there was a substantial proportion of outcome variance unexplained by the model predictors. Further studies are needed to investigate alternative factors, such as cultural attitudes or prior vaccine experiences, that may explain this variance and expand our understanding of the nocebo effect.

While vaccinations do cause some side effects, a proportion of vaccine-attributed side effects may be caused by psychologically associated nocebo effects. Specifically, anxiety, perceived sensitivity to vaccines, and prior symptom experiences increase the symptoms noticed following immunization. However, these factors as well as age and gender predispose an individual to misattribute benign symptoms as vaccine side effects. Future research would benefit from investigating whether approaches that address the nocebo effect can reduce the experience of vaccination side effects. The development of brief and clinically meaningful interventions is urgently needed to address persisting vaccine hesitancy and promote vaccine efforts.

## Data Availability

This study or analysis plan was not formally registered. De-identified data from this study are not available in a public archive. De-identified data from this study will be made available (as allowable according to institutional IRB standards) by emailing the corresponding author. The analytic code used to conduct the analyses presented in this study is not available in a public archive. They may be available by emailing the corresponding author. Materials used to conduct the study are not publicly available.
